# Oatmeal in Diabetes Mellitus

**Published:** 1908-11-07

**Authors:** 


### OATMEAL IN DIABETES MELLITUS.

It is a remarkable fact that although starch appears to be chemically the same substance whatever its source, there are clinical grounds for supposing that there must be differences in the ultimate compositions of starches from one source and starches from another. This possibility of there being minute but important chemical differences, at present undetectable by any known methods of analysis and yet of very real significance in medicine, is further suggested by the phenomena of such a disease as peripheral neuritis. Why should lead nearly always pick out the musculo-spiral nerve and select certain constant portions even of this, whereas the toxin of diphtheria shows a predilection for the palatal and ciliary nerves, and leprosy attacks sensory fibres, leaving the mingled motor strands quite untouched? These phenomena surely must depend upon definite but slight differences in the chemical composition of the various nerves.

Be this as it may, there seems to be good ground for supposing that oatmeal, though it is rich in carbohydrate, cannot only be well tolerated by some diabetics who cannot tolerate wheat-flour starch at all, but may even actually be of benefit to the patient.

It was Von Noorden who first pointed out this fact in 1902. Mosso had previously shown that some diabetics can take potatoes in abundance, although they are unable to eat wheat-flour. Either there are chemical differences between the starches of wheat, potatoes, and oatmeal respectively, or else there are different products associated with the starch in the three cases—the associated products in oatmeal benefiting some cases of diabetes mellitus, those in potatoes benefiting other cases of this disease, while wheat-starch nearly always has to be greatly limited or even eliminated from a diabetic dietary.

Von Noorden recommends quite large quantities of oatmeal—indeed, as much as the patient cares to take in the forms of porridge, oatcake and butter, oatmeal biscuits, oatmeal soup, and so on. It is impossible, however, to continue upon a full diet of oatmeal for many days at a time, partly because of the nausea and distaste that the patient develops, and partly because of the diarrhœa that is apt to arise.

It is well, therefore, to alternate the oatmeal with other diets; one or two days of abundant oatmeal may be followed by one or two when oatmeal is omitted and the meals are of the ordinary diabetic type. Then abundance of oatmeal in various forms for another day or two, and so on in a cycle.

If a case is not going to do well upon oatmeal the fact will become obvious very soon. In a favourable case, after a slight increase in the glycosuria for one or two days, the quantity of sugar begins to fall, and it soon comes down to a level similar to that obtained upon the strictest dieting. This is a very remarkable fact, and it would be equally remarkable even if it held good of only a small proportion of all cases. It has been proved to be true of quite a number of patients, young and old alike.

